# Epidemiology and Transmission Dynamics of Infectious Diseases and Control Measures

**DOI:** 10.3390/v14112510

**Published:** 2022-11-12

**Authors:** Sukhyun Ryu, June Young Chun, Sunmi Lee, Daesung Yoo, Yongdai Kim, Sheikh Taslim Ali, Byung Chul Chun

**Affiliations:** 1 Department of Preventive Medicine, Konyang University College of Medicine, Daejeon 35365, Republic of Korea; 2 Department of Internal Medicine, National Cancer Center, Goyang 10408, Republic of Korea; 3 Department of Applied Mathematics, Kyung Hee University, Yongin 17104, Republic of Korea; 4 Veterinary Epidemiology Division, Animal and Plant Quarantine Agency, Gimcheon 39660, Republic of Korea; 5 Department of Statistics, Seoul National University, Seoul 08826, Republic of Korea; 6 WHO Collaborating Centre for Infectious Disease Epidemiology and Control, School of Public Health, Li Ka Shing Faculty of Medicine, The University of Hong Kong, Hong Kong, China; 7 Laboratory of Data Discovery for Health Limited, Hong Kong Science and Technology Park, Hong Kong, China; 8 Department of Preventive Medicine, Korea University College of Medicine, Seoul 02841, Republic of Korea

**Keywords:** infectious disease, epidemiology, One Health, SARS-CoV-2, COVID-19, intervention

## Abstract

The epidemiology and transmission dynamics of infectious diseases must be understood at the individual and community levels to improve public health decision-making for real-time and integrated community-based control strategies. Herein, we explore the epidemiological characteristics for assessing the impact of public health interventions in the community setting and their applications. Computational statistical methods could advance research on infectious disease epidemiology and accumulate scientific evidence of the potential impacts of pharmaceutical/nonpharmaceutical measures to mitigate or control infectious diseases in the community. Novel public health threats from emerging zoonotic infectious diseases are urgent issues. Given these direct and indirect mitigating impacts at various levels to different infectious diseases and their burdens, we must consider an integrated assessment approach, ‘One Health’, to understand the dynamics and control of infectious diseases.

## 1. Introduction

Infectious diseases, including seasonal and emerging respiratory virus infections such as influenza and severe acute respiratory syndrome coronavirus 2 (SARS-CoV-2), have caused several epidemics and pandemics and significantly disrupted daily life. During the coronavirus 2019 (COVID-19) pandemic, we observed that public health and social measures (PHSMs) reduced the burden of the pandemic and other directly transmitted respiratory virus infections in many countries by reducing their transmissibility and susceptibility in the community [1,2,3,4,5,6,7]. Therefore, to reduce the overall burden of respiratory infections in the community, it is important to consider the direct and indirect impacts of PHSMs on diseases with similar transmission characteristics. Infectious disease epidemiology, a branch of epidemiology that studies why infectious diseases emerge and how they spread, can provide helpful information on developing and designing intervention strategies in different settings, from households (or small settings) to communities (or large settings). Such studies are often conducted by using and assimilating the syndromic, virological, and digital data, obtained from outbreak investigations, ongoing surveillance, and seroprevalence surveys. This data is enhanced with the advancement of statistical methods, mathematical modeling, and efficient computational tools. Herein, we explore an overview on the research scopes of developing epidemiological tools and possible applications for assessing the impact of PHSMs in the community and for infectious disease control with the necessity of the One Health paradigm, rather than review the specific technical details of methods.

## 2. Epidemiological Data Assimilation and Augmentation

**Figure 1:**
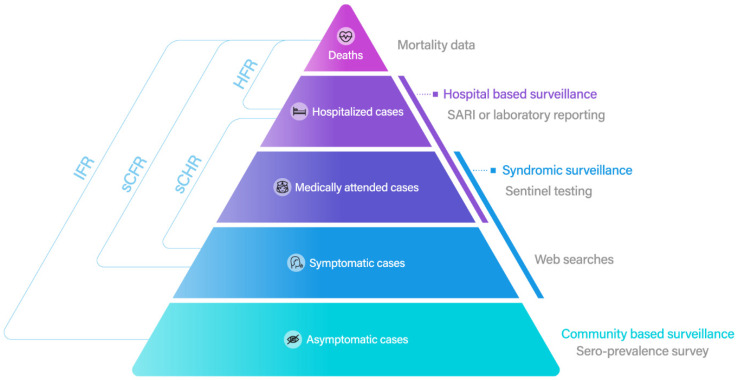
The burden pyramid of infectious disease and epidemiological severity parameters. The clinical spectrum of infectious diseases and the possible surveillance system at each level. Note: SARI = severe acute respiratory infection, IFR = infection fatality rate, sCFR = symptomatic cases fatality rate, sCHR = symptomatic hospitalization rate, HFR = hospitalization fatality rate.

The true epi-curves of any infectious disease are always challenging at the community level. The overall burden of these directly transmitted infectious diseases in the community starts with the infections, healthcare-seeking, hospitalization, severity (admission to the intensive care unit), and mortality from each disease (Figure 1). The syndromic information on the morbidity and mortality of these diseases is not free from biases and is often underestimated. We can only observe the tip of the ‘iceberg’ of total infections in the community. These data are the basis of epidemiological analyses, and the outcomes are highly data-driven. The improvements in data observation, surveillance systems, integration, and assimilation techniques, and data retrieval and reconstruction are crucial for understanding disease characteristics and transmission dynamics [3,8,9,10,11]. Therefore, correctly estimating the proportion of asymptomatic infections [12], identifying the case definitions over time [9], and nowcasting true infections [3,8,11] could lead to real-time epi-curves for any disease.

## 3. Inferring Epidemiological Parameters and Assessing the Impact of PHSMs

**Figure 2:**
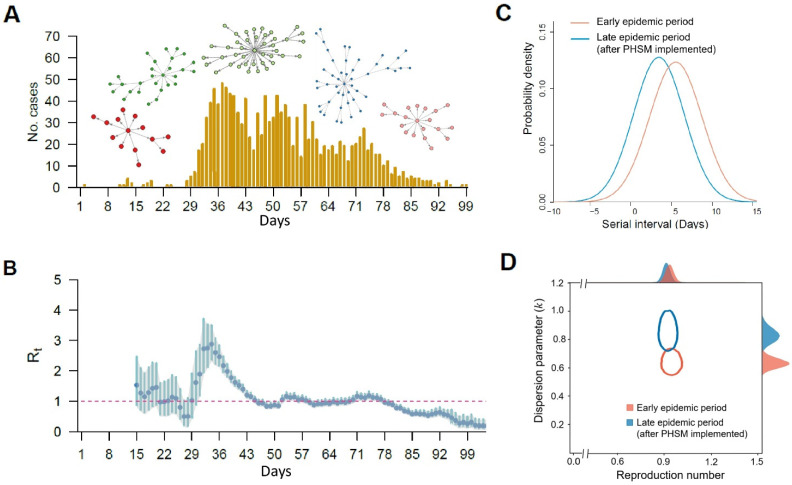
Epidemiological parameters to assess the impact of public health and social measures. (**A**) Epidemic curve with the transmission chain of the infectious diseases (a measure of transmission activity). (**B**) Daily effective reproductive number (*R_t_*) of the infectious diseases (a measure of transmissibility). (**C**) Serial interval distribution of the infectious diseases in two different epidemic periods. (**D**) Risk for superspreading events for infectious diseases during two different epidemic periods. Note: presented here are the outcomes of COVID-19 in South Korea, modified from [13,14].

Public health authorities often monitor the severity and status of an epidemic by assessing the transmission activity or/and the intensity (i.e., transmissibility) across the epidemic. For example, PHSMs, such as contract tracing, case isolations, hand hygiene, wearing a face mask, and social distancing, could affect people’s behavior and change the transmission activity or transmissibility of the underlining virus circulation in the community. The estimates of epidemiological parameters including the generation interval, effective reproductive number (*R_t_*), and superspreading potential (*k*) could reflect the transmission activity or the transmissibility of an epidemic and are used to infer the impact of these PHSMs (Figure 2).

The time-varying instantaneous reproduction number (*R_t_*) defines the average number of secondary cases generated from a typical primary case at time *t* (Figure 2B). The transmissibility of infectious diseases changes over time and is driven by several extrinsic factors, such as PHSMs and vaccination. Public health authorities widely use *R_t_* to assess the effectiveness of PHSMs and to inform their policy [15,16].

**Figure 3:**
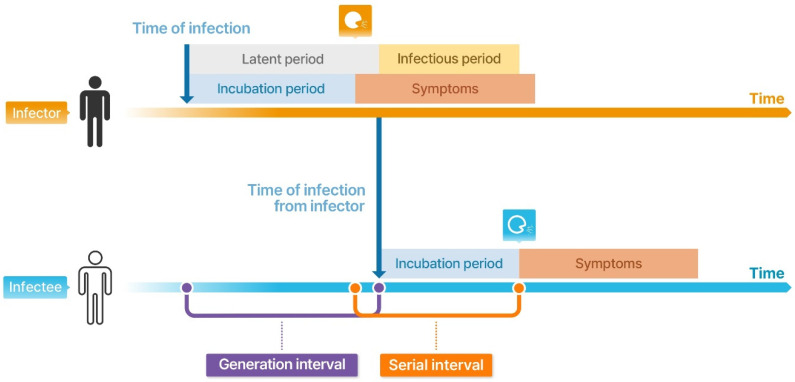
Schematic illustration of the generation interval and serial interval under biological and clinical processes. The latent period is the interval between the time of infection and when an individual becomes infectious, and the infectious period is the length of the time during which an individual can transmit a pathogen. The incubation period is the interval between the time of infection and the illness onset. The generation interval is the time between the infection events in successive cases in a transmission chain (i.e., between infector and infectee), and the serial interval is the time between the illness onset from infector to infectee.

The generation interval, the time between the infection events in successive cases in a transmission chain (Figure 3), is essential to estimating *R_t_*. However, the generation interval is difficult to infer with syndromic or clinical data since the infection process often goes unobserved. Therefore, it is usually approximated using a clinical measure, serial interval, the time between illness onset in successive cases in a transmission chain [15,17] (Figure 2C). The serial interval distribution is usually kept constant across the epidemic for a specific infectious disease, but recent studies report that it might change over time, accounting for the changes in PHSMs, such as case isolation and case profiles [17,18]. The time-varying estimate of effective serial interval distributions could improve the estimates of real-time *R_t_* and reduce the biases in using constant serial intervals over epidemics. This would better elucidate the effectiveness of PHSMs against disease transmission.

However, the *R_t_* and effective serial interval distribution do not reflect the individual variation of infectiousness. Therefore, the dispersion parameter (*k*) can be used to demonstrate the individual level of heterogeneity in transmission (Figure 2D). For example, a smaller *k* indicates the negative binomial distribution has a longer tail, meaning the individual variation in secondary cases is higher and the epidemic is more likely to be a superspreading event [19,20,21]. Furthermore, the estimate of the dispersion parameter may change over time due to the impact of PHSMs. Therefore, the time-varying dispersion parameter (*k_t_*) is proposed to account for the temporal changes in transmission heterogeneity at the individual level.

Post-COVID, many countries are relaxing PHSMs. However, there remains a significant knowledge gap regarding the association between the relaxation of PHSMs and changes in these epidemiological parameters [22]. Some studies reported that these parameters could potentially be reshaped for COVID-19 variants and subvariants [13,23] and would have an impact on other infectious disease dynamics. This could lead to an increase in the infection burden for future infectious diseases in different countries and settings [4,24,25].

## 4. An Improved Statistical Model with Age-Varying and Multi-Strain Susceptibility of Infections

During the COVID-19 pandemic, the pre-symptomatic transmission potential of SARS-CoV-2 hindered effective PHSMs. It is difficult to identify the transmission onset time as it is problematic to precisely determine who is infected and when. However, detailed contact-tracing exercises allow us to reconstruct transmission chains based on onset-time information, possibly using the infection timing with known potential exposure dates. Combining this data with known information regarding infector and infectee symptom onset, the incubation period and serial interval distributions can be inferred (Figure 3).

Infectious disease transmission depends on the number of infectious and susceptible individuals in the population and their effective contacts. For example, the stochastic susceptible–infectious–recovered compartmental model assumes a homogenous population and indicates the high likelihood of effective contact among identical age groups. All individuals in the population are not equally susceptible to infection. Therefore, information on age-specific infection, the probability of infection, and the contact matrix could be used to develop an age-structured compartment model [26,27]. For instance, early in the COVID-19 pandemic in 2020, few cases were reported among children [26], and an age-varying transmission matrix of infector–infectee pairs was observed (i.e., children had a higher probability of transmission to or from adults) [14]. Since public health authorities in many countries have reported an age-stratified number of COVID-19 cases daily, the age-specific force of infection (i.e., age-specific susceptibility of infection) could be estimated using the Bayesian inference method with the Markov chain Monte Carlo procedure [26,28,29].

Thus, the age-specific COVID-19 vaccination uptake and the effectiveness of vaccination can be applied to the susceptible population in an age-structured compartment model for COVID-19 [28,29]. In addition, many countries have changed their COVID-19 surveillance from an active to a passive scheme, and many COVID-19 cases have not been reported due to changes in health-seeking behaviors and a lack of contact tracing (Figure 1). Therefore, to identify the age-varying susceptibility of SARS-CoV-2 infection, seroprevalence studies have been conducted in many locations during the Omicron wave of SARS-CoV-2. In addition, a community longitudinal seroprevalence survey may improve epidemic modeling and pharmaceutical intervention strategies by elucidating the immune dynamic of SARS-CoV-2 at the individual and population levels.

Post-COVID-19, the co-circulation of multiple viruses in the community could address several crucial questions on cross-protection from natural infections due to similar viruses, the seasonality of multiple pathogens, and their integrated burden and impact on healthcare facilities. These create opportunities to develop a holistic quantitative approach.

## 5. Optimization of PHSMs Using Artificial Intelligence

The design of optimal intervention strategies is crucial to respond to epidemics or pandemics in real time. The optimization theory under optimal control problems is well-established [30,31]. However, optimization theory for individual-based models (or network models) has a relatively short history and is currently under development. Game theory can provide good methods for the optimization of individual-based models, while dynamic or geometric programming allows for the optimization of network-based models. The optimization problems (optimal control or dynamic programming) often require pre-determined information (priors) [30,31], and optimization frameworks using artificial intelligence (AI) provide an alternative approach to answering epidemiological questions. For example, it can be used to identify when to screen or treat infected individuals in a resource-limited setting and various infectious diseases (foot and mouth disease in animal infectious diseases [32] and human influenza in 2009 in England [33]).

AI-based research is a growing field, and has been applied in many sequential decision-making problems, such as playing Go Robot [34]. The AI-based reinforcement framework consists of two main entities, a policymaker (or an actor) and a dynamic model of disease transmission (or the environment). The actor makes a decision based on the contact network structure and information regarding the dynamically changing epidemiological characteristics of the population (susceptible–exposed–infectious–recovered). Reinforcement learning utilizes the past–present–future states and reveals inherited or embedded information. These features can provide an innovative and alternative framework for understanding the transmission dynamics of infectious diseases.

## 6. Importance of One Health for Future Zoonotic Infectious Disease Epidemics or Pandemics

Over the last decade, two coronavirus diseases, Middle East respiratory syndrome coronavirus and SARS-CoV-2, have affected the global population. It has been well documented that these zoonotic infectious diseases are transmitted from animal populations (e.g., wildlife and livestock) to susceptible humans or vice versa by direct or indirect contact [35]. This contact is more frequent owing to humans’ forceful encroachment into natural spaces due to the drastic growth of the human population [36] and traveling [37]. Moreover, climate and landscape changes have reshaped the interconnectivity between animals and humans, which has induced either the competition for or the allocation of natural resources [38]. This type of change in interconnectivity consistently occurs between wild and farmed animals because livestock are highly dependent on humans for breeding and feeding. In countries such as South Korea, many domestic ducks have acquired avian influenza from wildfowl [39,40]. Proactive surveillance for avian influenza in both wild and farmed birds have been conducted by animal health authorities. Furthermore, early detection and the depopulation of infected poultry flocks and the serosurveillance of farm workers have been conducted to prevent the possible spread of viruses from animals to humans (i.e., spillover) [41]. Therefore, an integrated and holistic approach such as ‘One Health’ could improve public health decision-making policies for community-based mitigation and intervention strategies against these infectious diseases [37].

## 7. Conclusions

A critical strategic breakthrough of PHSMs from a One Health perspective is needed to mitigate or control future infectious disease epidemics and pandemics. Continuous improvements in community-based epidemiological modeling and applied computational methods are required to provide scientific evidence to improve public health decision-making policies.

## Data Availability

Not applicable.
